# The Relationship between Depressiveness and Eating Behaviors among Women

**DOI:** 10.3390/nu16020195

**Published:** 2024-01-07

**Authors:** Kamila Rachubińska, Anna Maria Cybulska, Ewa Kupcewicz, Mariusz Panczyk, Barbara Ślusarska, Elżbieta Grochans, Daria Schneider-Matyka

**Affiliations:** 1 Department of Nursing, Pomeranian Medical University in Szczecin, 71-210 Szczecin, Poland; kamila.rachubinska@pum.edu.pl (K.R.); daria.schneider.matyka@pum.edu.pl (D.S.-M.); 2 Department of Nursing, Collegium Medicum, University of Warmia and Mazury in Olsztyn, 10-719 Olsztyn, Poland; ekupcewicz@wp.pl; 3 Department of Education and Research of Health Sciences, Faculty of Health Sciences, Medical University of Warsaw, Litewska 14/16 St., 00-518 Warsaw, Poland; mariusz.panczyk@wum.edu.pl; 4 Department of Family and Geriatric Nursing, Faculty of Health Sciences, Medical University of Lublin, Staszica 6 Street, 20-081 Lublin, Poland; barbara.slusarska@umlub.pl

**Keywords:** depression, depressiveness, eating disorders, orthorexia nervosa

## Abstract

(1) The objective of the study was to determine the relationship between depressiveness and the occurrence of eating disorders, i.e., emotional eating, uncontrolled eating, cognitive restraint of eating, and the risk of orthorexia. (2) The study was conducted among 556 women from the West Pomeranian Voivodeship (Poland). The study employed the diagnostic survey method using a questionnaire technique: The Beck Depression Inventory, the ORTO—15 Questionnaire, the Three-Factor Eating Questionnaire, and a sociodemographic questionnaire. (3) Higher depressiveness severity is associated with a higher score on the “Cognitive Restraint of Eating” scale. The authors’ original study demonstrated a statistically significant relationship only between depressiveness and the “Uncontrolled Eating” subscale (*p* = 0.001). (4) The results of this study suggest that depressiveness is an important factor that contributes to a better understanding of eating behaviors. In addition, the results of this study suggest that eating behaviors and psychological factors should be taken into account in psychological interventions in the treatment of eating disorders. The clinical goal can be considered to be an improvement in non-normative eating behaviors, such as a reduction in overeating episodes or eating less frequently in the absence of a feeling of hunger.

## 1. Introduction

Eating behaviors are determined by numerous factors, starting from climatic factors through to social and cultural as well as religious factors and ending with individual preferences or habits [1]. Food begins to fulfil many functions in human life, including a psychological one [2]. The perpetuation of the idea that foods, and sweets in particular, are a great reward or consolation can make adults start to use food as a tension reducer [3]. The feeling of discomfort or a problem is not dealt with in a manner appropriate for the situation but is solved with food instead [4]. The inability of individuals to control their own emotions and stress can have a negative impact on them [5]. Emotional eating involves increased consumption in response to both positive and negative emotions [6]. Strong emotions can reduce people’s control over how much food they eat. Unrestricted food consumption may disrupt eating habits, possibly leading to negative health consequences [7,8].

Eating disorders are defined as disorders of eating habits or weight control behaviors. Due to food restriction or overconsumption, dietary behaviors can cause significant somatic deterioration and even death. What is more, they impair psychosocial functioning, which may manifest itself in anxiety, depression, obsessive–compulsive disorder, self-aggression, decreased quality of life, disturbed self-image, reduced self-respect, and avoidance of social situations, particularly those involving eating food. Research has demonstrated the effect of eating disorders on the occurrence of depressiveness as well as the effect of depressiveness on the occurrence of eating disorders [9,10].

Eating disorders were first described among young women. The highest prevalence continues to be noted among girls at the peri-pubertal stage. Eating disorders, however, are also increasingly affecting men. Changes are also taking place in psychiatric classifications, as the diagnostic criteria previously in force are being modified, with new clinical syndromes being included. Knowledge of this issue is therefore important for pediatricians for a variety of reasons [11,12,13,14].

In recent years, there has been an increase in studies demonstrating a significant relationship between body image, eating habits, and emotional functioning in both clinical (e.g., patients with obesity or eating disorders) and non-clinical groups. The relevant literature provides different models considering the interrelationship between eating disorders and depressiveness. Some authors treat the occurrence of depressive disorders as secondary to eating disorders. Achieving symptomatic improvement is, in their opinion, expected to result in the resolution of depressiveness. Another hypothesis considers eating disorders to be a consequence of primary depressive disorders; in this interpretation, anorexia and bulimia nervosa are perceived as disorders masking the symptoms of depressiveness. It is, therefore, possible that depressiveness is secondary to eating disorders, and the symptoms of affective disorder often emerge following the occurrence of eating disorder symptoms and resolve with the resolution of the disease [15,16,17,18,19,20]. Concerning abnormal eating behaviors [21,22], three eating styles will be considered in the presented study: emotional eating, uncontrolled eating, and cognitive restraint of eating and orthorexia nervosa. Each of them contains a pathological aspect [23,24,25], i.e., (1) too frequent eating under the influence of emotions and stress (emotional eating) [26], (2) the loss of control over the amount of food consumed and the eating rate (uncontrolled eating) [27], and (3) unhealthy restriction of food intake (cognitive restraint of eating) [28].

It is estimated that 90–95% of people with eating disorders are women. The reasons for the female numerical predominance can be attributed to biological factors (significant fat gain and a change in body shape during puberty cause ambivalent feelings), psyche (orientation towards interpersonal relationships and emotions is linked to the need to be accepted, and appearance serves an important role in this respect), and culture (Western attractiveness patterns favoring a slim figure conflict with a consumerist lifestyle). Eating disorders associated with excessive food intake develop independently of initial body weight while often co-occurring with obesity, either accompanying it as a separate problem or directly leading to its development [10,29]. In addition, women are two to three times more likely to develop depression, which has been confirmed in numerous studies [30,31,32]. Female sex is one of the risk factors for depressiveness occurrence [33,34,35]. The objective of this study was to determine the relationship between depressiveness and the occurrence of eating disorders, i.e., too frequent eating under the influence of emotions and stress (emotional eating), the loss of control over the amount of food consumed and the eating rate (uncontrolled eating), and unhealthy restriction of food intake (cognitive restraint of eating) and the risk of orthorexia.

## 2. Materials and Methods

### 2.1. Organization and Course of the Study

The study involved 556 women from the West Pomeranian Voivodeship (Poland). The size of the study sample was calculated using Statistica, v. 13.1 (TIBCO Software Inc., StatSoft, Warszawa, Poland). A 95% confidence interval was assumed. This resulted in the sample being representative. According to the data on the female population in the West Pomeranian Voivodeship, the study should include at least 384 individuals. The criteria for inclusion in the study were as follows: age > 18 years, female sex, residence in the West Pomeranian Voivodeship, no diagnosed mental disorders, informed written consent to participate in the study, and the completion of the set of questionnaires. The presented study is part of a research project [36,37,38], approved by the Research Ethics Committee of the Pomeranian Medical University in Szczecin (KB-0012/518/12/16). The research tool sheets were distributed to women who read the above information and agreed to take part in the project. The respondents were informed about the purpose of the study and had the opportunity to ask questions and obtain explanations. The volunteers could withdraw from the study at any time without giving a reason. The time needed to complete the questionnaires was about 15 min.

### 2.2. Research Instruments

This survey-based study was performed using a questionnaire technique. The sociodemographic questionnaire and the following standardized tools adapted to the Polish conditions were used to analyze the relationship between depressiveness and eating disorders:

- The Beck Depression Inventory (BDI I-II) is used to measure the severity of depressive disorders. It includes 21 questions with answers rated on a four-point scale. The final scores are calculated by summing up the points obtained for each question. They reflect the level of depression and are interpreted as follows: 0–13: no depression or minimal symptoms of depression, 14–19: mild depression, 20–28: moderate depression, and 29–63: severe depression. BDI-II demonstrated adequate internal consistency at pre- and post-treatment (α = 0.87 and 0.90, respectively) [39,40].
- The ORTO-15 questionnaire, developed by Donini et al. [41], is the most popular research tool to measure the severity of orthorexia nervosa (ON) symptoms—the Orthorexia Risk Index (ORI). In the ORTO-15 test, a diagnosis of orthorexia is made for a score below the threshold that the test authors considered optimal, i.e., 40 points. The questions cover cognitive, emotional, and clinical aspects of ON. The validation of the ORTO-15 in Polish conditions was carried out by Janas-Kozi’s team. The value of Cronbach’s α statistics was satisfactory and was within the commonly accepted range of 0.7–0.9 [42].
- The Three-Factor Eating Questionnaire (TFEQ-13) is divided into three subscales measuring cognitive–behavioral and emotional aspects of eating behaviors. The first subscale measures behaviors related to restricting the quantity or type of food to control body weight and body image. The second one measures the tendency to eat more than usual due to loss of control over eating or an incontrollable feeling of hunger that triggers overeating binges. The third subscale measures overeating episodes caused by feelings of depressed mood and anxiety. The questions on the TFEQ-13 scale form three factors: (1) cognitive restraint of eating, (2) uncontrolled eating, and (3) emotional eating. These three factors reproduce 56.8% of the variability of the entire set of the observed variables. The Cronbach’s alpha internal consistency coefficient for the entire scale was 0.78, while for the subscales, it was 0.78, 0.76, and 0.72. The TFEQ-13 questionnaire contains standardized responses on a 4-point scale scored from 0 to 3 (definitely yes—3; rather yes—2; rather no—1; definitely no—0). Question 13 (R5) was re-coded as follows: 1 and 2—0; 3 and 4—1; 5 and 6—2; 7 and 8—3. The values are calculated separately for each subscale. No values are calculated for the total scale. The higher total score on a subscale indicates the severity of disorders within its range [43,44].
- The sociodemographic questionnaire included closed and semi-open-ended questions to acquire selected sociodemographic data for the respondents, i.e., age, educational background, marital status, residence, and professional activity.

### 2.3. Statistical Analysis

The data obtained from the study were analyzed using descriptive statistics techniques, which were chosen based on the measurement scale and variable type. For categorical variables, measured on a nominal or ordinal scale, data were displayed using structural indicators such as number (N) and frequency (%). For quantitative variables, which are measured on an interval scale, the following were employed: mean (M) and standard deviation (SD), median (Me), quartile range (Q1–Q2), and range (min–max).

To evaluate the differences in the mean values of the orthorexia risk index and the mean values for the three dimensions of cognitive–behavioral and emotional aspects of eating behaviors, i.e., (1) cognitive restraint of eating, (2) uncontrolled eating, and (3) emotional eating, depending on the level of depression (no depression or minimal symptoms of depression, mild depression, moderate depression, and severe depression), a one-way analysis of variance (ANOVA) with Fisher’s HSD post hoc test was employed.

The analyses also assessed the impact of selected factors (independent variables) on the following dependent variables: cognitive restraint of eating, uncontrolled eating, and emotional eating. For this purpose, single and multiple linear regression analyses were used. The independent variables were entered into the regression model simultaneously. For each independent variable, a standardized regression coefficient (β) with a 95% confidence interval (95% CI) was calculated. The parameter of the regression model was estimated using the least-squares method. Prior to the analyses, the following assumptions were verified: linearity, homoscedasticity, collinearity of independent variables, and normal distribution of residuals. The regression models also incorporated an evaluation of the mediation effect, wherein the BDI scoring variable served as the moderator.

Statistical analyses were conducted using Statistica™ 13.3 software (TIBCO Software, Palo Alto, CA, USA). A *p*-value below 0.05 was considered statistically significant.

## 3. Results

### 3.1. Sample

The study included 556 women from the West Pomeranian Voivodeship. In the statistical analyses, the mean values of the analyzed variables were, among others, as follows: the mean age of the respondents was 34 years. More than half of the respondents reported lower education (51.6%) and lived in a town with fewer than 100,000 inhabitants (52.3%). Most women were in a relationship (66.5%) and were employed (89.2%) [36,37,38] (Table S1).

### 3.2. Depression and Eating Behavior-Related Variables

The TFEQ-13 measures the cognitive–behavioral and emotional aspects of eating behavior. The questions of TFEQ-13 form three factors: cognitive restraint of eating, uncontrolled eating, and emotional eating. The mean score for cognitive restraint of eating was 6.21 ± 2.888. The mean score for uncontrolled eating was 5.59 ± 2.712. The mean score for emotional eating was 3.85 ± 1.552.

The data obtained via ORTO-15 measured the orthorexia risk index. The mean score was 37.45 ± 5.395. The respondents who scored more than 40 points, i.e., those who were in the orthorexia risk group, accounted for 38.1%. A higher total score on a subscale indicates the severity of disorders within its range.

The Beck Depression Inventory—BDI I-II measured the symptoms of depression. The mean score was 6.8 ± 7.345. Most women reported no depression subscale (85.1%), minimal symptoms of depression (7.7%), mild symptoms (5.2%), and moderate symptoms (2.0%) (Tables S2 and S3).

### 3.3. The Impact of Sociodemographic Variables and Severity of Depression on the Risk of Orthorexia

The obtained statistical results show that only age had a statistically significant effect on the risk of orthorexia. The scoring on the ORI scale increases with age (Table 1 and Table S4).

The effect of depressiveness severity on the ORI is not moderated by sociodemographic factors (Table S5). 

### 3.4. The Impact of Sociodemographic Variables and Severity of Depression on the Cognitive Restraint of Eating

The authors’ original study demonstrated a statistically significant relationship between depressiveness, marital status, age, and the “Cognitive Restraint of Eating” subscale (*p* < 0.001). Higher depressiveness severity was associated with a higher score on the “Cognitive Restraint of Eating” scale (Table 2). The individuals in a relationship had a significantly higher score on the “Cognitive Restraint of Eating” scale than single individuals (*p* = 0.038). The score on the “Cognitive Restraint of Eating” scale decreased with age (*p* < 0.001). Higher severity of depression symptoms was associated with a higher score on the “Cognitive Restraint of Eating” scale (Table 3). The effect of depressiveness severity on the score on the “Cognitive Restraint of Eating” scale was moderated by one sociodemographic variable, i.e., residence. In smaller towns (<100,000), the effect of depressiveness severity on cognitive restraint of eating was less pronounced than in larger towns (≥100,000) (*p* = 0.047) (Table 4).

### 3.5. The Impact of Sociodemographic Variables and Severity of Depression on Uncontrolled Eating

The authors’ original study demonstrated a statistically significant relationship only between depressiveness and the “Uncontrolled Eating” subscale (*p* = 0.001). Higher severity of depressiveness is associated with a higher score on the “Uncontrolled Eating” (*p* < 0.001) (Table 5). There was no effect of sociodemographic variables on the score on the “Uncontrolled Eating” scale (*p* > 001) (Table 6). The effect of depressiveness severity on the score on the “Uncontrolled Eating” scale was not moderated by sociodemographic factors (*p* > 0.01) (Table S6).

### 3.6. The Impact of Sociodemographic Variables and the Severity of Depression on Emotional Eating

No statistically significant relationship was found between the sociodemographic data, depressiveness, and the “Emotional Eating” subscale (*p* > 0.001) (Tables S7 and S8). The effect of depressiveness severity on the score on the “Emotional Eating” scale was not moderated by sociodemographic factors (*p* > 0.01) (Table S9).

## 4. Discussion

Eating disorders represent a significant health problem with a complex etiology, with them mainly affecting girls and young women, leading to numerous health, psychological, and social consequences [45,46]. This study was aimed at identifying relationships between depressiveness and the occurrence of eating disorders, namely too frequent eating under the influence of emotions and stress (emotional eating), loss of control over the amount of food consumed and the eating rate (uncontrolled eating), and the unhealthy restriction of food intake (cognitive restraint of eating) as well as the risk of orthorexia among women.

The authors’ study was conducted on a group of women. A study conducted among patients hospitalized with leukemia at the Świętokrzyskie Oncology Centre in Kielce showed that men, as compared to women, suppressed negative emotions more often. What is more, the lack of loved ones and loneliness may have exacerbated the feeling of anxiety [47]. This study confirms that men tended to suppress their emotions more than women. Social expectations of men not showing their feelings (understood as not showing one’s weakness) may contribute to higher scores in this regard in both healthy and ill individuals [8].

An analysis of previous research findings shows that depressiveness affects the perception of eating disorder symptoms [48,49], the assessment of cognitive, behavioral, and emotional functioning [50,51,52], and the severity of various psychopathological symptoms [53,54,55,56,57,58].

Based on the ORTO-15 questionnaire, it is possible to determine the risk of orthorexia occurrence in the women participating in the study. The authors’ study shows that, according to the Polish version, 38.1% (with a threshold of 40 points) of the respondents are in the orthorexia risk group. According to the results of a study by Bień et al., 71.43% of the respondents are in the risk group, while the results of a study by Donini et al. showed that 74.2% of the respondents were prone to orthorexia [41]. In a study by Ramacciotti et al., the prevalence of orthorexia among 1077 respondents was at a level of 57.6%, with the disorder twice as common in women than in men. These authors stress that these factors may help understand the importance of this disorder and primarily indicate the group of individuals that are more susceptible to its occurrence [59,60].

Studies conducted by Bağci Bosi et al. in 2007 and 2011 noted the prevalence of orthorexia in the Turkish population at levels of 45.5% and 57.6%, respectively [61]. On the other hand, in a study by Stochel, Janas-Kozik et al. [62], the prevalence of orthorexia in the Polish population was determined to be 13.7%. There is a large discrepancy between the study results, and this appears to be due not only to a different cut-off point adopted by the above-mentioned researchers (40 points in foreign analyses vs. 35 points in the study by Stochel, Janas-Kozik et al. and in this study). Nevertheless, a certain pattern can be observed, as studies undertaken currently show higher orthorexia prevalence than those conducted several years ago. This is probably related to the change of thinking about lifestyle and nutrition that is taking place in societies and the increasing awareness of nutrition and its impact on health [63,64].

The Public Opinion Research Center (CBOS) presented the results of a study entitled “Poles on their health and pro-health behaviour and activities”. These results show that more than 50% of the respondents consider healthy eating to be a pro-health measure [65,66,67].

Perhaps it also demonstrates the need to consider introducing orthorexia as a separate disease entity in the eating disorder group. The obtained statistical results show that only age had a statistically significant effect on the risk of orthorexia. Similar results were obtained in other studies [41,61,63]. Therefore, social factors appear to have a negligible effect on the occurrence of risk of orthorexia [64].

In addition to orthorexia, there has been an increase in the occurrence of eating disorders in recent years. The numerous factors contributing to this phenomenon include, e.g., the increase in the pace of life and the contribution of the mass media, which, on the one hand, promote a slim figure, while on the other hand, create a style of eating by promoting food advertisements [68,69]. Excessive focus on food and the resulting nutritional abnormalities can be the cause of many dangerous disorders and, consequently, result in pathological situations [70]. Eating disorders represent an important health problem with numerous health consequences, and the complex etiology of these disorders requires an interdisciplinary approach that considers, e.g., a modification of lifestyle, particularly eating behaviors, psychological support, or pharmacological treatment [46].

The concept of eating under the influence of emotions is derived from the psychosomatic theory of obesity, which assumes that obese people exhibit a greater tendency to eat triggered by experiencing emotions than people with normal body weight. In this case, episodic overeating is a response to negative feelings or stressful life events [71,72]. M. R. Yeomans et al. demonstrated that experiencing positive emotions also increased the tendency to overeat [73]. A dependence on food intake on emotional and psycho-affective needs often develops. The authors’ study showed no relationship between sociodemographic data, depressiveness, and the emotional eating subscale while demonstrating that higher depressiveness severity is associated with a higher score on the uncontrolled eating scale.

Anxiety and depression may lead to the excessive consumption of food as a means of coping, which leads to emotional and uncontrolled eating [74,75,76,77]. Pans et al. [78] revealed that the severity of depressive symptoms was associated with cognitive restraint of eating. The results of the authors’ study showed that higher depressiveness severity was associated with a higher score on the “Cognitive Restraint of Eating” scale. Individuals in a relationship have a higher cognitive restraint of eating level than single individuals. The score on the “Cognitive Restraint of Eating” scale decreases with age. In smaller towns (<100,000), the effect of depressiveness severity on the cognitive restraint of eating is less pronounced than that in larger towns (≥100,000).

By assumption, the use of dietary restrictions is intended to reduce body weight. However, due to the interplay between the factors described here (restrictions, eating under the influence of emotions, and the lack of control over eating), it is one of the main causes of body weight gain. Dietary restrictions can involve both limiting the quantity (energy content) and quality of meals, i.e., eliminating certain food components and eating only selected products [43].

A comparison of the mean results of the TFEQ-13 subscales by Dzielska et al. [43] showed that the total score in each subscale was related to the sex of the respondents and, in each case, was higher for women than for men. The higher scores for women on the food restriction scale may be linked to their greater tendency to go on weight-loss diets. In studies by other authors, the mean scores on this subscale were also higher for women than for men [79,80]. Analyses by J. C. Cappelleri et al. [80] indicate similar mean scores on the cognitive restraint of eating scale in both sexes. It should be noted that most of the research on eating behaviors has been conducted among women.

Effective disease prevention, active participation in the treatment process, and controlling one’s health requires both enhancing health competencies and developing psychosocial skills that are equated with health education and life skills [81].

## 5. Limitations and Implications for Professional Practice

The discourses on determining the relationships between depressiveness and the occurrence of eating disorders among Polish women presented in this study identified certain limitations and implications for professional practice. The main strength of our study was the use of important and reliable psychometric tools. The model did not test other potentially important predictors of the development of eating disorders, i.e., anxiety, stress, or insomnia. In addition, neither body weight nor BMI were addressed. Using larger samples from different socio-cultural contexts and the inclusion of other variables (e.g., income and social class) are some of the aspects that should be considered in the future. This limitation also represents the cross-sectional, correlational nature of the current project, with analyses based on the data collected, and limits the ability to identify causal relationships. A limitation of the use of the ORTO-15 questionnaire is the lack of results for the clinical group of individuals diagnosed as suffering from orthodoxia. Despite isolated descriptions of orthorexia [82,83], there are no accepted diagnostic criteria based on which it would be possible to establish a clinical group. It is difficult to clearly determine whether an individual scoring high on the questionnaire only represents a specific lifestyle associated with healthy eating or suffers from a pathological eating pattern [42]. Despite its limitations, this study provides important findings and can be a starting point for broader research into the effect of psychological factors, i.e., depressiveness, on the occurrence of eating disorders among Polish women and beyond. In theoretical terms, the current findings are important as they provide additional knowledge on the relationship between eating disorders and depressiveness. From a practical point of view, this study may have implications for therapists, as female sex appears to be a risk factor for the relationship between depressiveness and eating disorders. Mental health specialists should be aware of the relationship between depressiveness, eating disorders, and sex in order to implement appropriate prevention programs. There is a need to include measures aimed at identifying psychological factors contributing to the occurrence of eating disorders, i.e., too frequent eating under the influence of emotions and stress (emotional eating), loss of control over the amount of food consumed and the eating rate (uncontrolled eating), and the unhealthy restriction of food intake (cognitive restraint of eating) as well as orthorexia among women. It seems important to provide psychological support to people in need. It is also essential to take institutional preventive action to address the occurrence of psychological and eating disorders.

## 6. Conclusions

The results of this study suggest that depressiveness is an important factor that contributes to a better understanding of eating behaviors. In addition, the results of this study suggest that eating behaviors and psychological factors should be taken into account in psychological interventions in the treatment of eating disorders. The clinical goal can be considered to be an improvement in non-normative eating behaviors, such as a reduction in overeating episodes or eating less frequently without a feeling of hunger. In order to assist these individuals in their attempts to achieve healthy behaviors, variables related to mental functioning can then be identified as important goals to support individuals in their efforts to change their health behaviors by achieving better mental well-being. Relationships were demonstrated between depressiveness and the occurrence of uncontrolled Eating and cognitive restraint of eating. A higher depressiveness level was identified as an important predictor of higher results for uncontrolled eating and cognitive restraint of eating. Considering the sociodemographic data, it was also shown that age was a significant predictor of the ORI and cognitive restraint of eating. The cognitive restraint of eating level decreased with age. Individuals in a relationship had a higher cognitive restraint of eating score than single individuals. The effect of depressiveness severity on the cognitive restraint of eating scale score was moderated by one sociodemographic variable, i.e., residence. In smaller towns (<100,000), the effect of depressiveness severity on cognitive restraint of eating was less pronounced than in larger towns. The effect of depressiveness severity on the ORI, uncontrolled eating, and emotional eating was not moderated by sociodemographic factors.

## Figures and Tables

**Table 1:** A multivariate model—analysis of the effect of sociodemographic variables and the severity of depressiveness according to the BDI on the orthorexia risk occurrence according to ORTO-15.

	Level	ß	−95% CI	+95% CI	t	*p*
Absolute term					−0.910	0.363
Educational background	Primary + Vocational + Secondary (ref.)					
	Tertiary	−0.067	−0.154	0.021	−1.499	0.134
Residence	<100,000 (ref.)					
	≥100,000	−0.048	−0.132	0.037	−1.105	0.270
Marital status	Single (ref.)					
	In a relationship	−0.007	−0.095	0.081	−0.150	0.881
Professional activity	Inactive (ref.)					
	Active	−0.020	−0.112	0.072	−0.420	0.675
Age		0.102	0.006	0.198	2.091	0.037
BDI (scoring)		0.006	−0.077	0.090	0.149	0.882

ref.—reference level, ß—standardized regression coefficient, CI—confidence interval, and BDI—Beck Depression Inventory.

**Table 2:** A univariate model—analysis of the effect of sociodemographic variables and the severity of depressiveness according to the BDI on cognitive restraint of eating according to TFEQ-13.

	ß	−95% CI	+95% CI	t	*p*
Absolute term				28.001	<0.001
BDI (scoring)	0.224	0.143	0.306	5.421	<0.001

ß—standardized regression coefficient, CI—confidence interval, and BDI—Beck Depression Inventory.

**Table 3:** A multivariate model without moderation—analysis of the effect of sociodemographic variables and the severity of depressiveness according to the BDI on cognitive restraint of eating according to TFEQ-13.

	Level	ß	−95% CI	+95% CI	t	*p*
Absolute term					4.352	<0.001
Educational background	Primary + Vocational + Secondary (ref.)					
	Tertiary	0.009	−0.076	0.093	0.207	0.836
Residence	<100,000 (ref.)					
	≥100,000	0.055	−0.027	0.137	1.321	0.187
Marital status	Single (ref.)					
	In a relationship	0.091	0.005	0.176	2.084	0.038
Professional activity	Inactive (ref.)					
	Active	−0.046	−0.135	0.043	−1.008	0.314
Age		−0.166	−0.260	−0.073	−3.511	<0.001
BDI (scoring)		0.228	0.147	0.309	5.527	<0.001

ref.—reference level, ß—standardized regression coefficient, CI—confidence interval, and BDI—Beck Depression Inventory.

**Table 4:** A multivariate model with moderation—analysis of the effect of sociodemographic variables and the severity of depressiveness according to the BDI on cognitive restraint of eating according to TFEQ-13.

	ß	−95% CI	+95% CI	t	*p*
Absolute term				1.914	0.056
Marital status✻BDI	0.015	−0.147	0.176	0.178	0.859
Age✻BDI	−1.344	−6.233	3.545	−0.540	0.589
Professional activity✻BDI	−0.037	−0.236	0.162	−0.362	0.717
Educational background✻BDI	0.013	−0.145	0.171	0.165	0.869
Residence ✻BDI	0.153	0.002	0.305	1.994	0.047

ß—standardized regression coefficient, CI—confidence interval, BDI—Beck Depression Inventory, and ✻—moderation effect.

**Table 5:** A univariate model—analysis of the effect of sociodemographic variables and the severity of depressiveness according to the BDI on uncontrolled eating according to TFEQ-13.

	ß	−95% CI	+95% CI	t	*p*
Absolute term				26.734	<0.001
BDI (scoring)	0.139	0.056	0.221	3.295	0.001

ß—standardized regression coefficient, CI—confidence interval. and BDI—Beck Depression Inventory.

**Table 6:** A multivariate model without moderation—analysis of the effect of sociodemographic variables and the severity of depressiveness according to the BDI on uncontrolled eating according to TFEQ-13.

	Level	ß	−95% CI	+95% CI	t	*p*
Absolute term					2.699	0.007
Educational background	Primary + Vocational + Secondary (ref.)					
	Tertiary	−0.021	−0.107	0.066	−0.471	0.638
Residence	<100,000 (ref.)					
	≥100,000	−0.013	−0.097	0.071	−0.306	0.760
Marital status	Single (ref.)					
	In a relationship	0.003	−0.084	0.091	0.073	0.942
Professional activity	Inactive (ref.)					
	Active	−0.037	−0.129	0.054	−0.804	0.422
Age		−0.091	−0.187	0.004	−1.881	0.060
BDI (scoring)		0.137	0.054	0.220	3.230	0.001

ref.—reference level, ß—standardized regression coefficient, CI—confidence interval, and BDI—Beck Depression Inventory.

## Data Availability

The data presented in this study are available from the first author upon request.

## Supplementary Materials

The following supporting information can be downloaded at: https://www.mdpi.com/article/10.3390/nu16020195/s1. Table S1: General sociodemographic characteristics of the study group (N = 556). Table S2: Descriptive characteristics of variables in the study group of women; Table S3: Descriptive characteristics of variables in the study group of women; Table S4: A univariate model—Analysis of the effect of sociodemographic variables; Table S5: Analysis of the effect of sociodemographic variables and the severity of depressiveness according to BDI on the orthorexia risk occurrence according to ORTO-15; Table S6: A multivariate model with moderation—Analysis of the effect of sociodemographic variables and the severity of depressiveness according to BDI on Uncontrolled Eating according to TFEQ-13; Table S7: A univariate model—Analysis of the effect of sociodemographic variables and the severity of depressiveness according to BDI on Emotional Eating according to TFEQ-13; Table S8: A multivariate model without moderation—Analysis of the effect of sociodemographic variables and the severity of depressiveness according to BDI on Emotional Eating according to TFEQ-13; Table S9: A multivariate model with moderation—Analysis of the effect of sociodemographic variables and the severity of depressiveness according to BDI on Emotional Eating according to TFEQ-13.

## References

[1] Jeżewska-Zychowicz M. (2007). Zachowania Żywieniowe i Ich Uwarunkowania.

[2] Ogden J. (2011). Od zdrowych do zaburzonych zachowań żywieniowych. Psychologia Odżywiania Się.

[3] Bąk-Sosnowska M. (2010). Apetyt z emocji. Charaktery.

[4] Juruć A., Wierusz-Wysocka B., Bogdański P. (2011). Psychologiczne aspekty jedzenia i nadmiernej masy ciała. Farm Współ..

[5] Ziółkowska B. (2014). Psychospołeczne Aspekty Nienormatywnej Masy Ciała.

[6] Leszczyńska S., Błażejewska K., Lewandowska-Klafczyńska K., Rycielski P. (2011). Emocje a zachowania żywieniowe u kobiet w wieku 18-30 lat. Endokr. Otyłość. Zab. Przem Mat..

[7] Wieczorkowska-Wierzbińska G. (2011). Psychologiczne Ograniczenia.

[8] Stetkiewicz-Lewandowicz A., Wychowałek S., Sobów T. (2017). Emocjonalne aspekty zachowań żywieniowych. Probl. Hig. I Epidemiol..

[9] Nicolas I. (2008). Long-term evolution and complications of eating disorders. Rev. Prat..

[10] Bąk-Sosnowska M. (2010). Zaburzenia odżywiania towarzyszące otyłości. Forum Zaburzeń Metab..

[11] Pilecki M.W., Śmierciak N. Co Pediatra Powinien Wiedzieć o Zaburzeniach Odżywania Się. https://www.researchgate.net/publication330970261_Co_pediatria_powinien_wiedziec_o_zaburzeniach_odzywania_sie_What_should_a_pediatrician_know_about_eating_disorders,.

[12] American Psychiatric Association (1994). Diagnostic and Statistical Manual of Mental Disorders.

[13] Krawczyk P., Święcicki Ł. (2020). ICD-11 vs. ICD-10-przegląd aktualizacji i nowości wprowadzonych w najnowszej wersji Międzynarodowej Klasyfikacji Chorób WHO. Psychiatr. Pol..

[14] Kupfer D.J., First M.B., Regier D.A. (2008). A research agenda for DSM V.

[15] Boutelle K.N., Braden A., Knatz-Peck S., Anderson L.K., Rhee K.E. (2018). An open trial targeting emotional eating among adolescents with overweight or obesity. Eat. Disord..

[16] Claudat K., Lavender J.M. (2018). An introduction to the special issue on emotion regulation and eating disorders. Eat. Disord..

[17] Fuller-Tyszkiewicz M., Richardson B., Lewis V., Smyth J., Krug I. (2018). Do women with greater trait body dissatisfaction experience body dissatisfaction states differently? An experience sampling study. Body Image.

[18] Haynos A.F., Wang S.B., Fruzzetti A.E. (2018). Restrictive eating is associated with emotion regulation difficulties in a non-clinical sample. Eat. Disord..

[19] Pellizzer M.L., Waller G., Wade T.D. (2018). Body image flexibility: A predictor and moderator of outcome in transdiagnostic outpatient eating disorder treatment. Int. J. Eat. Disord..

[20] Racine S.E., Horvath S.A. (2018). Emotion dysregulation across the spectrum of pathological eating: Comparisons among women with binge eating, overeating, and loss of control eating. Eat. Disord..

[21] Allirot X., Miragall M., Perdices I., Baños R.M., Urdaneta E., Cebolla A. (2017). Effects of a Brief Mindful Eating Induction on Food Choices and Energy Intake: External eating and mindfulness state as moderators. Mindfulness.

[22] Grimm E.R., Steinle N.I. (2011). Genetics of eating behavior: Established and emerging concepts. Nutr. Rev..

[23] Conceição E.M., Utzinger M., Pisetsky J.E. (2015). Eating disorders and problematic eating behaviours before and after bariatric surgery: Characterization, assessment and association with treatment outcomes. Eur. Eat. Disord. Rev..

[24] Stellpflug C. (2010). Emotional Eating “The Battle for the Mind”!. http://www.k12academics.com/articles/emotional-eating-%E2%80%9C-battle-mind-%E2%80%9D#.VuBIhZzhDIU.

[25] Strien van T., Ouwens M.A., Engel C., Weerth de C. (2014). Hunger, inhibitory control and distress-induced emotional eating. Appetite.

[26] Alberts H.J., Thwissen R., Raes L. (2012). Dealing with problematic eating behaviour. The effects of a mindfulness-based intervention on eating behaviour, food cravings, dichotomous thinking and body image concern. Appetite.

[27] Verzijl C.L., Ahlich E., Schlauch R.C., Rancourt D. (2018). The role of craving in emotional and uncontrolled eating. Appetite.

[28] Brytek-Matera A., Czepczor-Bernat K., Modrzejewska A. (2021). Związek między zachowaniami żywieniowymi, obrazem ciała a dysregulacją emocjonalną: Podobieństwa między osobami z nadmierną i prawidłową masą ciała. Psychiatr. Pol..

[29] Simon R.W., Lively K. (2010). Sex, anger and depression. Soc. Forces..

[30] Sloan D.M., Sandt A.R. (2006). Gender differences in depression. Womens Health.

[31] Burke R., Oberklaid F., Burgess Z. (2004). Workaholism among Australian women psychologists: Antecedents and consequences. Women Manag. Rev..

[32] Chuick C.D., Greenfeld J.M., Greenberg S.T., Shepard S.J., Cochran S.V., Haley J.T. (2009). A qualitative investigation of depression in men. Psychol. Men Masc..

[33] Kessler R.C., Berglund P., Demler O., Jin R., Koretz D., Merikangas K.R., Rush A.J., Walters E.E., Wang P.S., National Comorbidity Survey Replication (2003). The epidemiology of major depressive disorder: Results from the National Comorbidity Survey Replication (NCS-R). JAMA.

[34] Kuehner C.C. (2003). Gender differences in unipolar depression: An update of epidemiological findings and possible explanations. Acta. Psychiatr. Scand..

[35] Marek K., Białoń P., Wichowicz H., Melloch H., Nitka-Siemińska A. (2005). Przesiewowa ocena rozpowszechnienia objawów depresyjnych i lękowych wśród studentów Akademii Medycznej w Gdańsku. Psychiatria.

[36] Rachubińska K., Cybulska A.M., Kupcewicz E., Jurewicz A., Panczyk M., Cymbaluk-Płoska A., Grochans E. (2022). Loneliness and the Degree of Addiction to Shopping and Work among Polish Women: The Mediating Role of Depression. J. Clin. Med..

[37] Rachubińska K., Cybulska A., Szkup M., Grochans E. (2021). Analysis of the relationship between personality traits and Internet addiction. Eur. Rev. Med. Pharmacol. Sci..

[38] Rachubińska K., Cybulska A.M., Owsianowska J., Śniegocka M., Zair L., Grochans E. (2022). The relationship between women’s personality traits and addiction to social networking sites on the example of Facebook. Eur. Rev. Med. Pharmacol. Sci..

[39] Titov N., Dear B.F., McMillan D. (2011). Psychometric comparison of the PHQ-9 and BDI-II for measuring response during treatment of depression. Cogn. Behav. Ther..

[40] Zawadzki B., Popiel A., Pragłowska E. (2009). Charakterystyka Psychometryczna Polskiej Adaptacji Kwestionariusza Depresji BDI-II Aarona, T. Becka. Psychol. Etiol. Genet..

[41] Donini L.M., Marsili D., Graziani M.P., Imbriale M., Cannella C. (2004). Orthorexia nervosa: A preliminary study with a proposal for diagnosis and an attempt to measure the dimension of the phenomenon. Eat. Weight Disord..

[42] Stochel M., Janas-Kozik M., Zejda J.E., Hyrnik J., Jelonek I., Siwiec A. (2015). Walidacja kwestionariusza ORTO-15 wgrupie młodzieży miejskiej wwieku 15–21 lat. Psychiatr. Pol..

[43] Dzielska A., Mazur J., Małkowska-Szkutnik A., Kołoło H. (2009). Adaptacja polskiej wersji kwestionariusza Three-Factor Eating Questionnaire (TFEQ-13) wśród młodzieży szkolnej w badaniach populacyjnych. Probl. Hig. I Epidemiol..

[44] Stunkard A.J., Messick S. (1985). The three-factor eating questionnaire to measure dietary restraint, disinhibition and hunger. J. Psychosom. Res..

[45] Bień A., Pieczykolan A. (2017). Eating disorders among women of childbearing age. J. Educ. Health Sport.

[46] Rzońca E., Bień A., Iwanowicz-Palus G. (2016). Zaburzenia odżywiania–problem wciąż aktualny. Eating disorders—An ongoing problem. J. Educ. Health Sport.

[47] Cieślik A., Marmurowska-Michałowska H. (2004). Poziom kontroli emocji u chorych na białaczki. Ann. UMCS.

[48] Wiatrowska A.K. (2021). Nasilenie objawów i struktura depresji u kobiet z zaburzeniami odżywiania. Lub. Rocz. Pedagog..

[49] Bizeul C., Brun J.M., Rigaud D. (2003). Depression influences the EDI scores in anorexia nervosa patients. Eur. Psychiatry.

[50] Giel K.E., Wittorf A., Wolkenstein L., Klinberg S., Drimmer E., Schönenberg M., Rapp A.M., Fallgatter A.J., Hautzinger M., Zipfel S. (2012). Is impaired set-shifting a feature of pure anorexia nervosa? Investigating the role of depression in set-shifting ability in anorexia nervosa and unipolar depression. Psychiatry Res..

[51] Hambrook D., Oldershaw A., Rimes K., Schmidt U., Tchanturia K., Treasure J., Richards S., Chalder T. (2011). Emotional expression, self-silencing, and distress tolerance in anorexia nervosa and chronic fatigue syndrome. Br. J. Clin. Psychol..

[52] Pilska M., Jeżewska-Zychowicz M. (2008). Psychologia Żywienia.

[53] Hudson J.I., Hiripi E., Pope H.G., Keddler R.C. (2007). The prevalence and correlates of eating disorders in the national comorbidity survey replication. Biol. Psychiatry.

[54] Fisher S., Le Grange D. (2007). Comorbidity and high-risk behaviors in treatment-seeking adolescents with bulimia nervosa. Int. J. Eat. Disord..

[55] Wiatrowska A. (2019). Podmiotowe Korelaty Samooceny Kobiet z Zaburzeniami Odżywiania.

[56] Rajewski A., Namysłowska I. (2012). Zaburzenia odżywiania. Psychiatria Dzieci i Młodzieży.

[57] Kucharska K., Wilkos E., Jarema M. (2016). Zaburzenia odżywiania. Psychiatria. Podręcznik dla Studentów Medycyny.

[58] Brytek-Matera A., Brytek-Matera A. (2020). Emocje i ich regulacja a jedzenie: Wzajemne współzależności. Psychodietetyka.

[59] Vargas M., Thege B.K., Dukay-Szabó S., Túry F., van Furth E.F. (2014). When eating healthy is not healthy: Orthorexia nervosa and its measurement with the ORTO-15 in Hungary. BMC Psychiatry.

[60] Ramacciotti C.E., Perrone P., Coli E., Burgalassi A., Conversano C., Massimetti G., Dell’Osso L. (2011). Ortorexia nervosa in the general population: A preliminary screening using a self-administered questionnaire (ORTO-15). Eat. Weight. Disord. Stud. Anorex. Bulim. Obes..

[61] Bağci Bosi A.T., Camur D., Güler C.B. (2007). Prevalence of orthorexia nervosa in resident medical doctors in the faculty of medicine (Ankara, Turkey). Appetite.

[62] Janas-Kozik M., Zejda J., Stochel M., Brożek G., Janas A., Jelonek I. (2012). Ortoreksja—Nowe rozpoznanie?. Psychiatr. Pol..

[63] Hyrnik J., Janas-Kozik M., Stochel M., Jelonek I., Siwiec A., Rybakowski J.K. (2016). The assessment of orthorexia nervosa among 1899 Polish adolescents using the ORTO-15 questionnaire. Int. J. Psychiatry Clin. Pract..

[64] Łucka I., Domarecki P., Janikowska-Hołoweńko D., Plenikowska-Ślusarz T., Domarecka M. (2019). The prevalence and risk factors of orthorexia nervosa among school-age youth of Pomeranian and Warmian-Masurian voivodeships. Psychiatr. Pol..

[65] Komunikat z Badań CBOS (2012). Polacy o Swoim Zdrowiu Oraz Prozdrowotnych Zachowaniach i Aktywnościach.

[66] Gubiec E., Stetkiewicz-Lewandowicz A., Rasmus P., Sobów T. (2015). Problem ortoreksji w grupie studentów kierunku dietetyka. Med. Ogólna I Nauk. O Zdrowiu..

[67] Jagielska G., Wolańczyk T., Osuch B. (2010). Zaburzenia miesiączkowania w jadłowstręcie psychicznym. Psychiatr. Pol..

[68] Kędra E., Pietras J. (2011). Zaburzenia odżywiania—Znak naszych czasów. Probl. Hig. I Epidemiol..

[69] Kałędkiewicz E., Doboszyńska A. (2013). Ortoreksja na tle innych zaburzeń odżywiania. Forum Med. Rodz..

[70] Nowogrodzka A., Piasecki B. (2012). Zaburzenie odżywiania—różnice międzypłciowe. Now. Lek..

[71] Arnow B., Kenardy J., Agras W.S. (1995). The Emotional Eating scale: The development of a measure to assess coping with negative effects by eating. Int. J. Eat. Disord..

[72] Tylka J. (2000). Otyłość w ujęciu psychosomatycznym. Psychosomatyka. Wybrane Zagadnienia z Teorii i Praktyki.

[73] Yeomans M.R., Coughlan E. (2009). Mood-induced eating: Interactive effects of restraint and tendency to overeat. Appetite.

[74] Yau Y.H.C., Potenza M.N. (2019). Stress and eating behaviors. Minerva Endocrinol..

[75] Andersen J.R., Aasprang A., Bergsholm P., Sletteskog N., Våge V., Natvig G.K. (2010). Anxiety and depression in association with morbid obesity: Changes with improved physical health after duodenal switch. Health Qual. Life Outcomes.

[76] Boutelle K.N., Hannan P., Fulkerson J.A., Crow S.J., Stice E. (2010). Obesity as a prospective predictor of depression in adolescent females. Health Psychol..

[77] Aoun C., Nassar L., Soumi S., El Osta N., Papazian T., Rabbaa Khabbaz L. (2019). The cognitive, behavioral, and emotional aspects of eating habits and association with impulsivity, chronotype, anxiety, and depression: A cross-sectional study. Front. Behav. Neurosci..

[78] Paans NP G., Bot M., Brouwer I.A., Visser M., Roca M., Kohls E. (2018). The association between depression and eating styles in four European countries: The MooDFOOD prevention study. J. Psychosom. Res..

[79] Karlsson J., Persson L.O. (2000). Psychometric properties and factor structure of the Three-Factor Eating Questionnaire (TFEQ) in obese men and women. Results from the Swedish Obese Subjects (SOS) study. Int. J. Obes..

[80] Cappelleri J.C., Bushmakin A.G. (2009). Psychometric analysis of the Three-Factor Eating Questionnaire-R21: Results from a large, diverse sample of obese and non-obese participants. Int. J. Obes..

[81] Woynarowska-Sołdan M., Kozłowska-Wojciechowska M. (2017). Zachowania żywieniowe i aktywność fizyczna pracowników szkół oraz działania dla poprawy tych zachowań i ich efekty. Hygeia.

[82] Bratman S., Knight D. (2000). Health Food Junkies: Orthorexia Nervosa: Overcoming the Obsession with Healthful Eating.

[83] Zamora M.L.C., Bonaechea B.B., Sanchez F.G., Rial B.R. (2005). Orthorexia nervosa. A new eating disorder?. Actas. Espan. Psiquiatr..

