# Supplementary material for: The Relationship between Depressiveness and Eating Behaviors among Women
Source: Nutrients. 2024 Jan 7;16(2):195. doi: 10.3390/nu16020195 (PMC10821179; doi:10.3390/nu16020195)
Supplement: Supplementary file 1 [file nutrients-16-00195-s001.zip › nutrients-2750119-supplementary.pdf]

Table S1. General sociodemographic characteristics of the study group (N = 556).

Sociodemographic variables		n	%
**Education**	Lower (secondary, vocational, elementary)	287	51.6
	Higher	269	48.4
Marital status	Single (maiden, divorced, widow)	186	34.5
	in a formal/informal relationship	370	66.5
Place of residence	< 100.000 inhabitants	293	52.7
	≥ 100.000 inhabitants	263	47.3
Professional activity	Professionally active	496	89.2
	Professionally inactive	60	10.8

n – number of cases, % – percentage of the total study group

Table S2. Descriptive characteristics of variables in the study group of women

Variable	M	SD	Me	Q1–Q3	Min – Max
Emotional Eating	3.85	1.55	4.0	3.0 – 5.0	0.0 – 9.0
Uncontrolled Eating	5.59	2.71	5.5	4.0 – 7.0	0.0 – 15.0
Cognitive Restraint of Eating	6.21	2.89	6.0	4.0 – 8.0	0.0 – 15.0
ORI	37.45	5.40	38.0	35.0 – 41.0	15.0 – 55.0
BDI (scoring)	6.80	7.35	4.5	4.5 – 10.0	0.0 – 40.0

M – mean, SD – standard deviation, Me – median, Q1 – lower quartile, Q3 – upper quartile, Min – minimum, Max – maximum, ORI – Orthorexia Risk Index, BDI – Beck Depression Inventory

Table S3. Descriptive characteristics of depressive symptoms according to BDI I–II and the risk of orthorexia according to ORTO–15

BDI I–II	n	%
no depression	473	85.1
minimal symptoms of depression	43	7.7
mild depression	29	5.2
moderate depression	11	2.0
ORTO–15		
ORI	344	61.9
No risk of orthorexia	212	38.1

ORI – Orthorexia Risk Index, BDI – Beck Depression Inventory

severity of depressiveness according to BDI on the orthorexia risk occurrence according to ORTO–15

Table S4. A univariate model – Analysis of the effect of sociodemographic variables

	ß	–95% CI	+95% CI	t	p
Absolute term				37.692	< 0.001
BDI (scoring)	0.007	–0.077	0.090	0.159	0.874

ß – standardised regression coefficient, CI – confidence interval, BDI – Beck Depression Inventory

Table S5. Analysis of the effect of sociodemographic variables and the severity of depressiveness according to BDI on the orthorexia risk occurrence according to ORTO–15

	**ß**	**−95% CI**	**+95% CI**	**t**	**p**
Absolute term				0.016	0.988
Marital status ✻ BDI	−0.001	−0.168	0.166	−0.012	0.991
Age ✻ BDI	1.795	−3.258	6.847	0.698	0.486
Professional activity ✻ BDI	0.133	−0.073	0.338	1.269	0.205
Educational background ✻ BDI	−0.051	−0.215	0.112	−0.619	0.536
Residence ✻ BDI	0.061	−0.096	0.217	0.762	0.446

ß – standardised regression coefficient, CI – confidence interval, BDI – Beck Depression Inventory

✻ moderation effect

Table S6. A multivariate model with moderation – Analysis of the effect of sociodemographic variables and the severity of depressiveness according to BDI on Uncontrolled Eating according to TFEQ–13

	**ß**	**−95% CI**	**+95% CI**	**t**	**p**
Absolute term				1.087	0.277
Marital status ✻ BDI	0.038	−0.128	0.203	0.450	0.653
Age ✻ BDI	−1.339	−6.363	3.685	−0.524	0.601
Professional activity ✻ BDI	−0.133	−0.337	0.072	−1.277	0.202
Educational background ✻ BDI	0.040	−0.122	0.203	0.489	0.625
Residence ✻ BDI	0.054	−0.101	0.210	0.687	0.492

ß – standardised regression coefficient, CI – confidence interval, BDI – Beck Depression Inventory

✻ moderation effect

Table S7. A univariate model – Analysis of the effect of sociodemographic variables and the severity of depressiveness according to BDI on Emotional Eating according to TFEQ–13

	**ß**	**−95% CI**	**+95% CI**	**t**	**p**
Absolute term				32.272	< 0.001
BDI (scoring)	0.073	−0.011	0.156	1.714	0.087

ß – standardised regression coefficient, CI – confidence interval, BDI – Beck Depression Inventory

Table S8. A multivariate model without moderation – Analysis of the effect of sociodemographic variables and the severity of depressiveness according to BDI on Emotional Eating according to TFEQ–13

	**Level**	**ß**	**−95% CI**	**+95% CI**	**t**	**p**
Absolute term					2.317	0.021
Educational background	Primary+Vocational+Secondary (ref.)					
	Tertiary	0.019	−0.068	0.106	0.428	0.669
Residence	< 100,000 (ref.)					
	≥ 100,000	−0.033	−0.117	0.052	−0.752	0.452
Marital status	single (ref.)					
	in a relationship	0.051	−0.037	0.140	1.141	0.255

	Level	ß	−95% CI	+95% CI	t	p
Professional activity	inactive (ref.)					
	active	0.009	−0.083	0.101	0.197	0.844
Age		−0.067	−0.163	0.030	−1.361	0.174
BDI (scoring)		0.078	−0.006	0.161	1.821	0.069

ß – standardised regression coefficient, CI – confidence interval, BDI – Beck Depression Inventory

Table S9. A multivariate model with moderation – Analysis of the effect of sociodemographic variables and the severity of depressiveness according to BDI on Emotional Eating according to TFEQ−13

	ß	−95% CI	+95% CI	t	p
Absolute term				1.097	0.273
Marital status✻BDI	−0.139	−0.305	0.027	−1.644	0.101
Age✻BDI	−0.822	−5.867	4.223	−0.320	0.749
Professional activity✻BDI	−0.177	−0.382	0.029	−1.690	0.092
Educational background✻BDI	0.127	−0.036	0.290	1.531	0.126
Residence✻BDI	−0.035	−0.191	0.121	−0.443	0.658

ß – standardised regression coefficient, CI – confidence interval, BDI – Beck Depression Inventory

✻ moderation effect
